# Association between alterations in plasma metabolome profiles and laminitis in intensively finished Holstein bulls in a randomized controlled study

**DOI:** 10.1038/s41598-021-92163-6

**Published:** 2021-06-17

**Authors:** Sonja Christiane Bäßler, Ákos Kenéz, Theresa Scheu, Christian Koch, Ulrich Meyer, Sven Dänicke, Korinna Huber

**Affiliations:** 1 Institute of Animal Science, University of Hohenheim, 70599 Stuttgart, Germany; 2 Department of Infectious Diseases and Public Health, City University of Hong Kong, Hong Kong, SAR China; 3 Educational and Research Centre for Animal Husbandry, Hofgut Neumuehle, 67728 Muenchweiler a.d. Alsenz, Germany; 4 Institute of Animal Nutrition, Friedrich-Loeffler-Institut (FLI), Federal Research Institute for Animal Health, 38116 Brunswick, Germany

**Keywords:** Chronic inflammation, Metabolic diseases, Metabolomics

## Abstract

Metabolic consequences of an energy and protein rich diet can compromise metabolic health of cattle by promoting a pro-inflammatory phenotype. Laminitis is a common clinical sign, but affected metabolic pathways, underlying pathophysiology and causative relationships of a systemic pro-inflammatory phenotype are unclear. Therefore, the aim of this study was to elucidate changes in metabolome profiles of 20 months old Holstein bulls fed a high energy and protein diet and to identify novel metabolites and affected pathways, associated with diet-related laminitis. In a randomized controlled feeding trial using bulls fed a high energy and protein diet (HEP; metabolizable energy [ME] intake 169.0 ± 1.4 MJ/day; crude protein [CP] intake 2.3 ± 0.02 kg/day; calculated means ± SEM; n = 15) versus a low energy and protein diet (LEP; ME intake 92.9 ± 1.3 MJ/day; CP intake 1.0 ± 0.01 kg/day; n = 15), wide ranging effects of HEP diet on metabolism were demonstrated with a targeted metabolomics approach using the AbsoluteIDQ p180 kit (Biocrates Life Sciences). Multivariate statistics revealed that lower concentrations of phosphatidylcholines and sphingomyelins and higher concentrations of lyso-phosphatidylcholines, branched chain amino acids and aromatic amino acids were associated with an inflammatory state of diet-related laminitis in Holstein bulls fed a HEP diet. The latter two metabolites share similarities with changes in metabolism of obese humans, indicating a conserved pathophysiological role. The observed alterations in the metabolome provide further explanation on the underlying metabolic consequences of excessive dietary nutrient intake.

## Introduction

Application of intensive feeding regimens is a common practice in beef cattle production systems, in order to promote growth performance. However, chronic excessive dietary nutrient intake (energy via starch and protein) is known to increase the risk of developing metabolic disorders in animals, as well as in humans. In humans, these disorders, such as insulin resistance and dyslipidemia, are commonly labelled as obesity-related morbidities, or as clusters of the human metabolic syndrome^1^. In companion animals, similar concepts have been established for horses, i.e. the equine metabolic syndrome in relation to a starch-rich diet and sedentary lifestyle^2^. In farm animals, fattening lambs developed insulin resistance and metabolic disorders during the fattening period, particularly when fed restrictively during early life^3^. In fattening bulls, an energy rich finishing diet before slaughter is traditionally accepted as a method to achieve production targets. These diets include protein- and starch-rich components. However, excessive intake of starch-rich and low fiber diets are also known to induce proinflammatory alterations, such as rumen acidosis, tail tip necrosis or laminitis in cattle^4–6^. These diseases are commonly referred to as production diseases and affect health and performance of the animals and thus also animal welfare.

Dietary interventions such as protein-rich diets and experimental fructose overload were presented as major factors for laminitis^7–9^, which was in the focus of our study. Laminitis is one of the clinically apparent signs of the intensive feeding related metabolic disorders in cattle, with lesions including yellow waxy discolorations, hemorrhages of the sole, separation of the white line or erosion of heel^10^, and ridging of the dorsal claw wall^11^. Laminitis was also identified as a major cause for lameness in dairy cows^12^. However, it is still poorly understood what metabolic pathways might be affected by intensive feeding that eventually lead to clinically apparent inflammatory conditions such as laminitis. Laminitis is known to be a multifactorial disease, being a key symptom of metabolic disorders. Blood analyses of affected cows showed indications of inflammation and metabolic acidosis^8^, but did not provide more detailed insights into which pathways or metabolites are involved.

Metabolomics technologies provide a snapshot of the concentrations of a wide array of metabolites in a biological sample. As these metabolites are intermediary or end products of various metabolic pathways, this provides a good indication of the current metabolic condition. With the AbsoluteIDQ p180 Kit (Biocrates Life Sciences AG, Innsbruck, Austria), new biomarkers and affected pathways were successfully found in metabolic disorders like human diabetes^13^, insulin dysregulated horses^14^ and retained placenta in dairy cows^15^. This kit was established to identify metabolites of several pathways associated with changes in energy metabolism, amino acid metabolism, pro-inflammatory signaling, dysregulation of glucose and lipid metabolism, obesity-related disorders, insulin resistance, and mitochondrial dysfunction.

The major objective of this study was to investigate metabolic consequences of an experimental diet, comprising a chronically increased nutrient (energy, starch and protein) intake delivered by additional dietary concentrate feed, in comparison to a feeding regimen only based on forage. Currently, our understanding about how these dietary factors modulate metabolic pathways is incomplete. We hypothesized that excessive intake of energy and protein provokes alterations of metabolism, reflected by quantifiable changes in the plasma metabolome, leading to a pro-inflammatory phenotype, clinically apparent as laminitis. Therefore, the aim of this study was to elucidate changes in metabolite profiles of Holstein bulls fed a high energy and protein diet (HEP), based on forage and a high level of concentrate versus bulls fed a low energy and protein diet (LEP), based on only forage. Further, we aimed to identify novel metabolites and associated metabolic pathways linked to a diet-related inflammatory phenotype reflected by laminitis. The outcome of this study allowed us (1) to characterize metabolic phenotype determined by a chronic intake of high energy and protein diet and associated with laminitis, and (2) to generate new hypothesis about the metabolic relationships between a high energy and protein intake and a systemic inflammatory phenotype for further mechanistic studies.

## Results

Ingredients and chemical composition of the TMR are given in Tables 1 and 2, respectively. Data of feed intake, body weight and average daily gain (ADG) during experimental time is shown in Table 3. Intake of DM, average metabolizable energy (ME), crude protein (CP) and starch (CS) were on average greater in HEP than in LEP (means ± SEM; DM: 14.8 ± 0.1 kg/day vs 9.2 ± 0.1 kg/day, P < 0.001; ME: 169.0 ± 1.4 MJ ME/day vs 92.9 ± 1.3 MJ ME/day, P < 0.001; CP: 2.3 ± 0.02 kg/day vs 1.0 ± 0.01 kg/day, P < 0.001; CS: 4.4 ± 0.04 kg/day vs 1.1 ± 0.02 kg/day, P < 0.001). Means of ADG during the experimental period were greater in HEP (1474 ± 45 g/day) than LEP (957 ± 53 g/day, P < 0.001) and HEP bulls were on average more than 100 kg heavier at slaughter.

**Table 1:** Ingredients of total mixed rations (TMR) fed during the experimental period.

Ingredients of TMR^a^		
	Feeding group	
Component, % of DM	HEP	LEP
Grass silage	40.33	69.99
Corn silage	13.68	29.92
Concentrate feed (including minerals)	45.99	0
Salt	0	0.09

^a^*DM* dry matter, *HEP* high energy and protein diet, *LEP* low energy and protein diet.

**Table 2:** Chemical composition of total mixed rations (TMR) fed during the experimental period.

Chemical composition of TMR^a^		
	Feeding group	
Item, g/kg of DM unless noted otherwise	HEP	LEP
DM, g/kg	510	369
ME, MJ/kg of DM	11.4	10.2
Crude protein	155.0	110.0
Crude fat	40.0	37.0
Crude fiber	171.0	239.0
aNDFom	356.0	428.0
ADFom	174.0	232.0
Ash	82.0	85.0
Total sugar	31.0	20.0
Total starch	297.0	123.0
Ca	6.6	5.2
P	3.6	2.7
Na	1.4	1.0
K	18.8	24.1
Mg	2.2	2.1

^a^*DM* dry matter, *ME* metabolizable energy, *aNDFom* neutral detergent fibre assayed with a heat stable amylase and expressed exclusive of residual ash, *ADFom* acid detergent fibre expressed exclusive of residual ash, *HEP* high energy and protein diet, *LEP* low energy and protein diet.

**Table 3:** Feed intake, body weight and average daily gain during experimental period.

Means per group/month^A^	Feed intake kg dry matter/day/animal		Body weight kg		Average daily gain g/day	
Month of life	HEP	LEP	HEP	LEP	HEP	LEP
14	13.9 ± 0.7^h,i^	7.2 ± 0.5^1,i^	527 ± 10.1^h,i^	501 ± 7.9^i^	1040 ± 424^d^	134 ± 407^f^
15	15.4 ± 0.6^f,g^	9.4 ± 0.5^h,i^	579 ± 11.1^f,g^	518 ± 8^h,i^	1883 ± 353^a^	590 ± 435^e^
16	15.5 ± 0.5^d,e^	9.4 ± 0.5^g,h^	633 ± 10.6^d,e^	552 ± 8.4^g,h^	1841 ± 229^a^	1198 ± 384^b,c,d^
17	15.6 ± 0.8^c,d^	9.9 ± 0.5^f,g^	676 ± 10.2^c,d^	581 ± 9^f,g^	1620 ± 228^a^	1077 ± 236^d^
18	14.1 ± 1.2^b,c^	9.4 ± 0.6^e,f^	710 ± 10.3^bc^	609 ± 9.5^e,f^	1149 ± 285^d^	970 ± 234^d,e^
19	13.9 ± 0.3^b^	10.3 ± 0.1^d,e^	753 ± 11.5^b^	652 ± 11^d,e^	1615 ± 471^a,b^	1573 ± 372^a,b,c^
20	13.1 ± 1.0^a^	10.2 ± 0.6^bc^	807 ± 9.4^a^	712 ± 11.5^bc^	1168 ± 338^c,d^	1156 ± 194^c,d^

^A^Means ± SD; ^a,b,c,d,e,f,g,h,i^levels not connected by the same letter are significantly different (P < 0.05). Effect of time P < 0.001, group P < 0.001, time × group P < 0.001; with the exception of body weight: effect of time × group P < 0.01.

**Figure 1:**
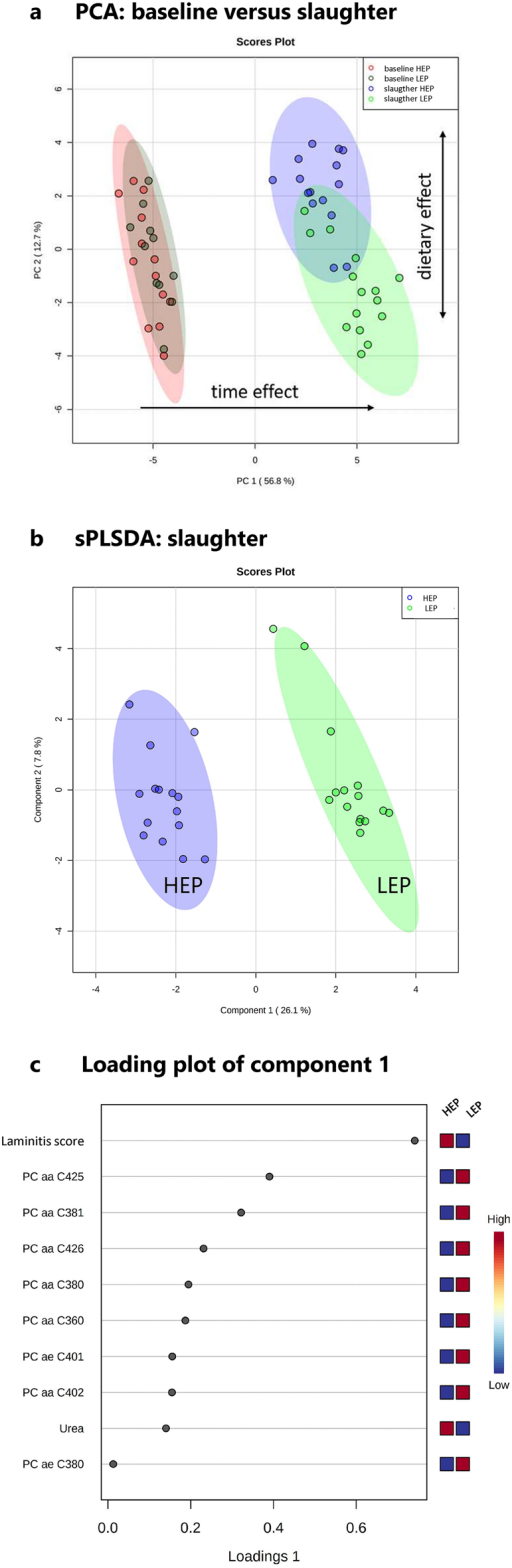
Exploratory data analysis. (**a**) Principal component analyses (PCA) of the combined data set of blood metabolome, body weight, insulin concentration, laminitis score, heel horn erosion and interdigital dermatitis score. Group fed low energy and protein diet (LEP): baseline n = 11, slaughter n = 15. Group fed high energy and protein diet (HEP): baseline n = 13, slaughter n = 15. Each dot represents the data of one bull at one sampling day. (**b**) Scores plot of sparse partial least squares-discriminant analysis (sPLS-DA) between components 1 and 2 applied to the dataset only at slaughter, n = 15 (blood metabolome, blood biochemistry, insulin concentration, body weight, laminitis score, heel horn erosion and interdigital dermatitis score). (**c**) Loadings of component 1 calculated by sPLS-DA of the dataset at slaughter. The variables listed on the left were ranked by the absolute values of their loadings, the color scheme on the right shows relative differences of the compounds between the feeding groups at slaughter. PC x:y = Phosphatidylcholine with total length of x carbon atoms and y unsaturated bonds in its acyl chains, aa = diacyl, ae = acyl-alkyl.

To visualize how dietary treatments affected plasma metabolome and clinical status along the experimental period, results were presented by principal component analysis to reduce the dimensionality of the data set and enhance interpretability (Fig. 1a). Dietary groups had similar profiles at baseline, but a clear time-dependent shift along principal component (PC) 1 and strong separation between the feeding groups along PC2 were observed by the end of the trial. In order to demonstrate the dietary effects in more detail, Fig. 1b presents a scores plot of sparse partial least squares-discriminant analysis (sPLS-DA), showing the results of both feeding groups at the time of slaughter. The compounds contributing the most to the overall variance between feeding groups at slaughter are shown in Fig. 1c, by the loadings of component 1 of sPLS-DA. Most prominent was the laminitis score. Furthermore, 8 phosphatidylcholines (PC) with different length and urea concentration were listed. Classification error rates (cross validation) of sPLS-DA showed 0% error rates for the first 3 components and 3.3% for components 4 and 5, indicating that the fit of the sPLS-DA model was appropriate.

### Claw disease

**Figure 2:**
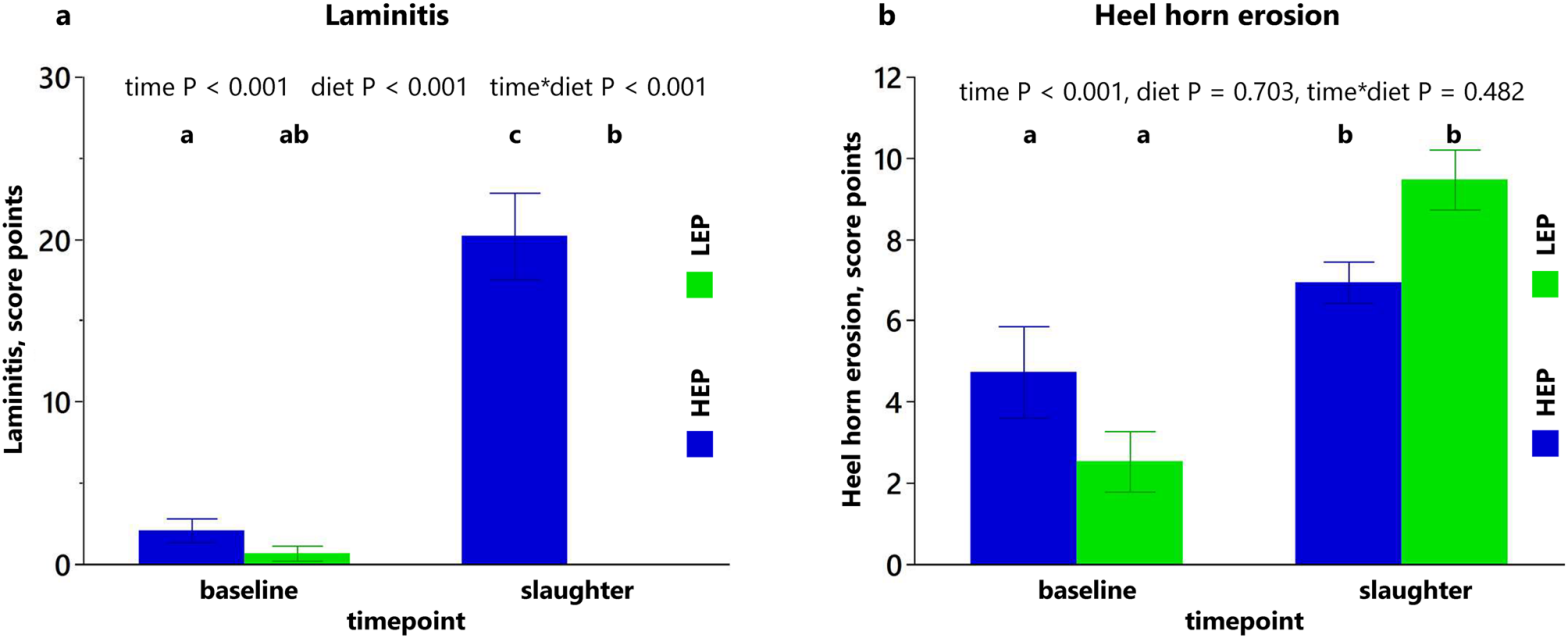
Claw disease. (**a**) Score points of laminitis and (**b**) score points of heel horn erosion at baseline and slaughter. Data shown as means ± SEM, n = 15, different letters indicate significant difference (P < 0.05). HEP = group fed high energy and protein diet, LEP = group fed low energy and protein diet.

Prevalence of claw disease at baseline and slaughter is presented in Table 4. None of the animals had digital dermatitis, tyloma, or interdigital phlegmon during the feeding trial. In both groups, occurrence of sole ulcers at typical and atypical localizations was less frequent at slaughter, while heel horn erosion and interdigital dermatitis occurred more frequently at slaughter. Only laminitis was highly influenced by dietary treatment, all HEP animals, but none of LEP, had laminitis at slaughter. Evaluating the grade of the most frequently occurred disease, score points of laminitis and heel horn erosion are shown in Fig. 2. Occurrence and severity of laminitis (Fig. 2a) increased over time only in HEP. Occurrence and severity of heel horn erosion (Fig. 2b) increased over time with no diet effects. Length of the dorsal claw wall was influenced by the diet, HEP having longer claw walls than LEP at slaughter (Table 4). The average thickness of the sole horn was reduced over time in both groups (Table 4).

**Table 4:** Claw disease and size at the beginning of the trial (baseline, 13 months of age) and at slaughter (20 months of age).

Timepoint	Baseline		Slaughter		Effect summary P values		
Dietary treatment group	HEP	LEP	HEP	LEP	Time	Diet	Time × diet
**Affected animals per group %**							
Sole ulcer, atypical	33^a^	40^a^	0^b^	0^b^	< 0.001	1.000	1.000
Sole ulcer, typical	47^a^	60^a^	7^b^	0^b^	< 0.001	0.347	0.175
Laminitis	40^a^	13^ab^	100^c^	0^b^	1.000	< 0.001	< 0.001
Heel horn erosion	67^a^	60^a^	100^b^	100^b^	< 0.001	1.000	1.000
Dermatitis interdigitalis	0^a^	0^a^	27^ab^	47^b^	< 0.001	1.000	1.000
Dermatitis digitalis	0	0	0	0			
Tyloma	0	0	0	0			
Interdigital phlegmon	0	0	0	0			
**Length of the dorsal claw wall**^A^							
Digit cranial medial	8.1 ± 0.7^b^	8.3 ± 0.7^a,b^	8.9 ± 0.9^a^	7.7 ± 0.6^b^	0.650	< 0.05	< 0.001
Digit cranial lateral	8.2 ± 0.6^a,b^	8.2 ± 0.5^a,b^	8.7 ± 0.7^a^	7.8 ± 0.8^b^	0.922	< 0.01	< 0.05
Digit caudal medial	8.3 ± 0.6^a,b^	8.3 ± 0.5^a,b^	8.6 ± 1.0^a^	7.7 ± 0.7^b^	0.340	< 0.05	< 0.05
Digit caudal lateral	7.9 ± 0.5^b^	8 ± 0.6^a,b^	8.5 ± 0.8^a^	7.5 ± 0.6^b^	0.801	< 0.01	< 0.001
Thickness of the sole horn^A^ (average of all digits)	0.7 ± 0.1	0.7 ± 0.1	0.6 ± 0.1	0.6 ± 0.2	< 0.05	0.611	0.279

^A^Means ± SD, n = 15. Values in cm; ^a,b,c^Levels not connected by the same letter are significantly different (P < 0.05).

### Carcass performance

**Figure 3:**
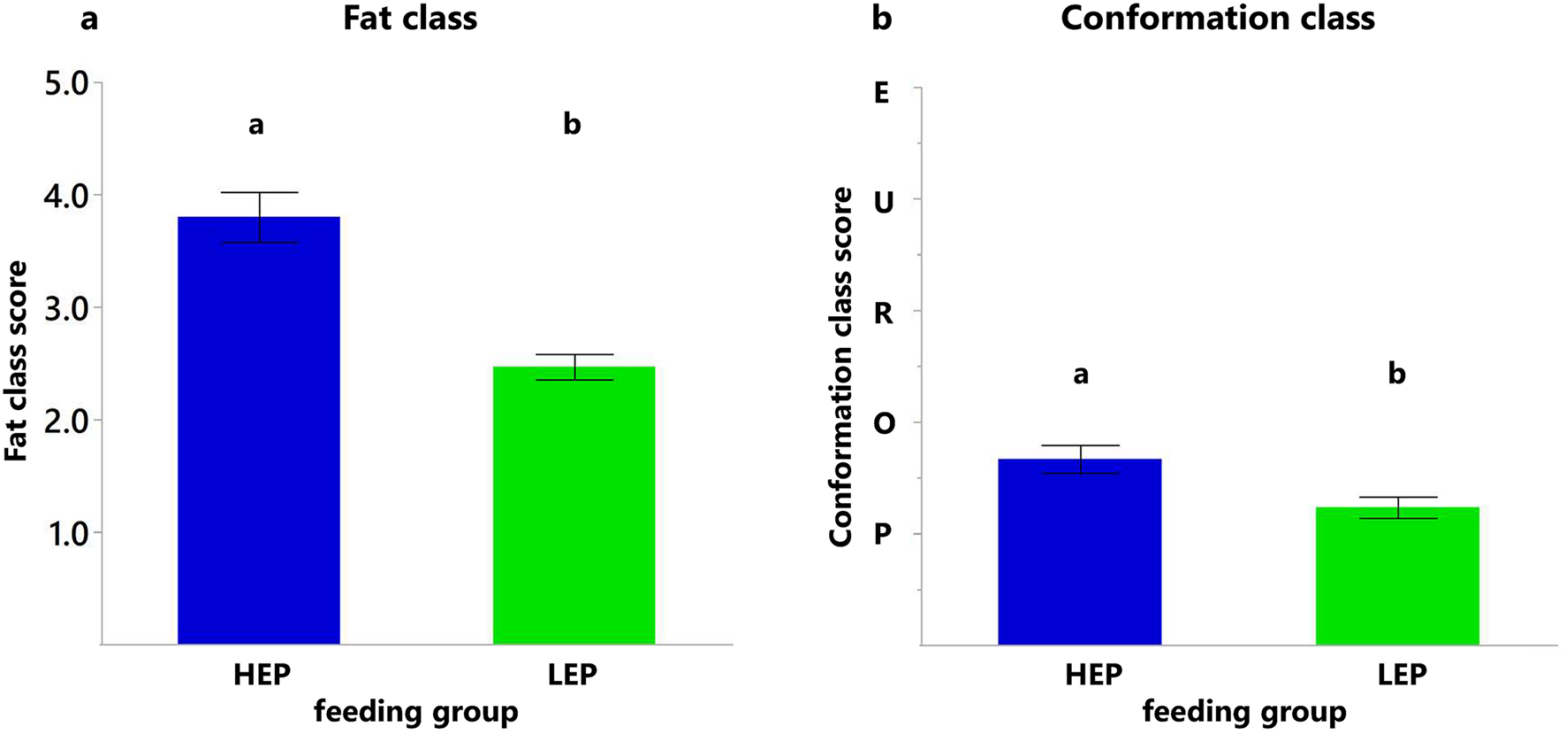
Carcass performance. (**a**) Fat class and (**b**) conformation class at slaughter. Data shown as means ± SEM, n = 15, different letters indicate significant difference (P < 0.05). HEP = group fed high energy and protein diet, LEP = group fed low energy and protein diet.

Carcasses of HEP showed higher fat and conformation classes than of LEP (Fig. 3a,b). Fat class was higher by 1.3 classes, and conformation class was higher by 0.4 classes in HEP.

### Insulin and glucose profiles

**Figure 4:**
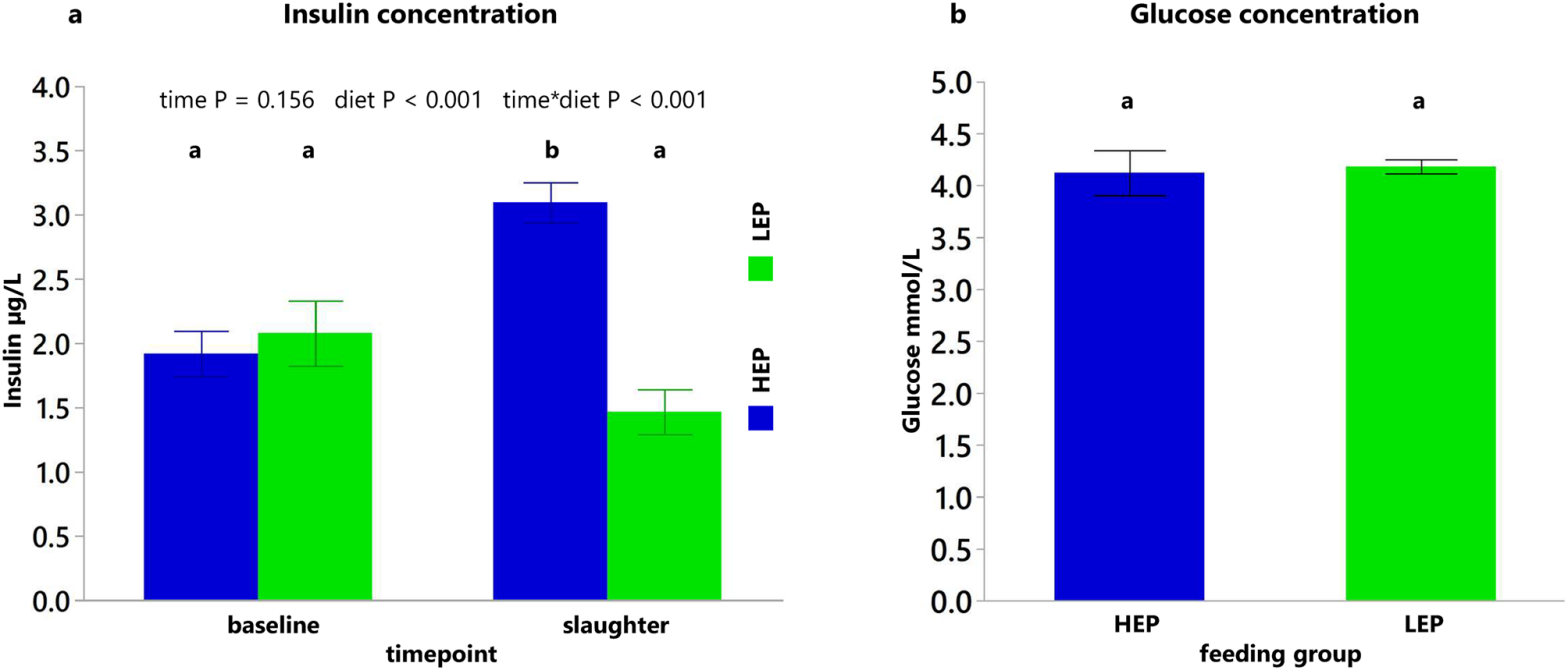
Insulin and glucose profile. (**a**) Insulin concentration in serum at baseline and slaughter. (**b**) Glucose concentration in plasma at slaughter. Data shown as means ± SEM, n = 15, different letters indicate significant difference (P < 0.05). HEP = group fed high energy and protein diet, LEP = group fed low energy and protein diet.

The effect on circulating insulin and glucose concentration is presented in Fig. 4. Insulin concentration was influenced by diet and interaction of time × diet. At slaughter, HEP had 2 times greater insulin concentration than LEP (Fig. 4a). At the same time, glucose concentration was not influenced by the diet (Fig. 4b, P = 0.794). Consequently, glucose-to-insulin ratio at slaughter was lower in HEP bulls, 1.22 ± 0.08 mmol/10^−3^ g, compared to LEP, 2.95 ± 0.31 mmol/10^−3^ g (mean ± SEM; P < 0.001).

### Metabolome profiles

**Figure 5:**
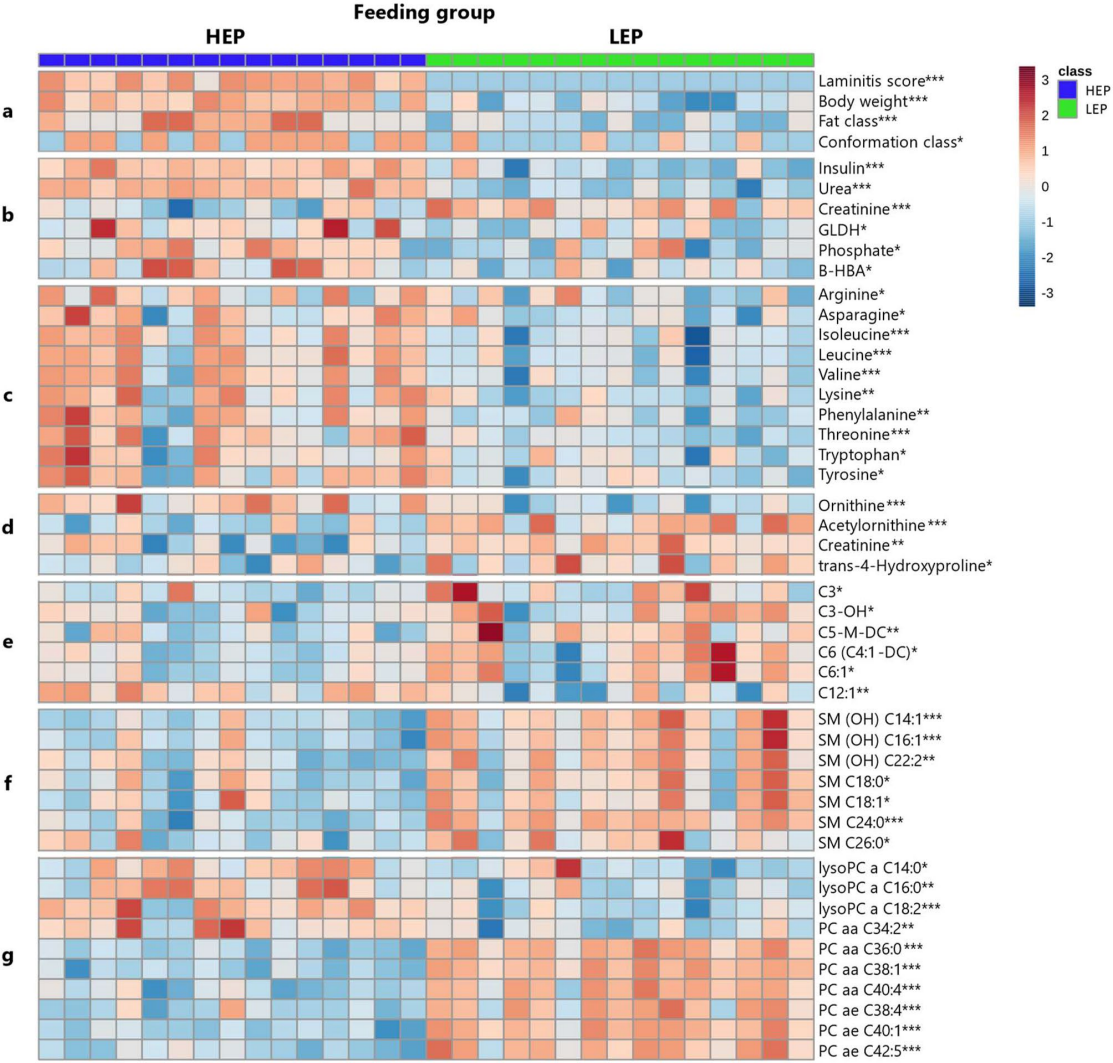
Heatmap visualizing all differentiating metabolites of interest at slaughter of bulls fed high energy and protein (HEP) and low energy and protein (LEP) diet, including (**a**) claw disease and growth performance, (**b**) insulin and blood biochemistry, (**c**) amino acids, (**d**) amino acids related, (**e**) acylcarnitines, (**f**) sphingomyelins (SM), (**g**) lyso-phosphatidylcholines (PC), PC aa 34:2 and 6 representative diacyl (aa) and acyl-alkyl (ae) PC. Each column represents one animal’s metabolite profile. All significantly different results are shown in Supplementary Table S1, n = 15, *P < 0.05, **P < 0.01, ***P < 0.001.

Figure 5 presents an overview of the metabolites of interest significantly differentiating between HEP and LEP fed bulls, calculated by Euclidean cluster analysis and visualized in a heatmap. Features were grouped according to their substrate class. In all features, feeding groups expressed a specific metabolite profile with high inter-individual variation. Figure 5a shows the distribution of claw diseases and growth performance. Figure 5b presents insulin and blood biochemistry. Only 5 out of 14 analyzed parameters of blood biochemistry showed a diet effect; namely urea, beta-hydroxybutyrate (BHB), glutamate dehydrogenase (GLDH) and phosphate, which were greater in HEP, and creatinine, which was lower in HEP. Concentrations of glucose, lactate, total protein, albumin, gamma-glutamyltransferase, aspartate aminotransferase, creatine kinase and non-esterified fatty acids did not differ between the feeding groups. A complete set of all blood biochemistry variables is listed in Supplementary Table S1. The substrate class of amino acids is demonstrated in Fig. 5c, 11 of them were greater in HEP including the 3 branched chain amino acids (BCAA) valine, leucine and isoleucine, the 3 aromatic amino acids (AAA) phenylalanine, tyrosine and tryptophan, as well as arginine, asparagine, threonine and lysine. Figure 5d shows the non-proteinogenic amino acid ornithine, which was greater in HEP, and 3 amino acid derivates acetylornithine, creatinine and trans-4-hydroxyproline, which were lower in HEP. Furthermore, 6 acylcarnitines were influenced by the diet (Fig. 5e), 5 short-chain acylcarnitines were lower in HEP, only dodecenoylcarnitine (C12:1) was greater in HEP. Figure 5f presents 7 sphingomyelins (SM), which were all lower in the HEP group. Especially the substrate class of glycerophospholipids (Fig. 5g) was strongly influenced by the diet. Sixty of 76 measured PCs were significantly different between the feeding groups: the majority of them was lower in HEP, while the concentrations of PC aa 34:2 and of 3 lyso-PCs were greater in HEP (Fig. 5g). Diacyl (aa) and acyl-alkyl (ae) PCs showed an overall homogeneous pattern, so in Fig. 5g only 6 PCs are presented, representing the majority of PCs. All significantly different metabolites are listed in Supplementary Table S1.

## Discussion

Our study demonstrated clear differences in metabolite profiles of Holstein fattening bulls, associated with an intensive feeding regimen consisting of increased starch and protein supply and with laminitis. Intensive fattening translates into promotion of daily weight gain and muscle fullness of carcass by chronic and excessive nutrient intake. However, in the present study the two feeding levels (HEP vs LEP) were chosen at experimentally high and experimentally low levels in order to provoke clear changes in metabolism. According to the guidelines of the Bavarian State Research Center for Agriculture (LfL)^16^, the protein and starch content of the HEP diet was above the recommended level, while metabolizable energy was still within the recommended range. In contrast, the metabolizable energy and protein and starch levels were below the recommended range in the LEP ration. The effects of the dietary regimens can only be evaluated as a whole, and testing interactions between feed components was not possible.

The HEP bulls showed higher daily weight gains and final live weights than the LEP group, consistent with other studies which compared different fattening intensities^4,17^. The increased energy and protein intake led to a greater muscle fullness, but even more clearly to an adiposity of the animals. This could indicate that the 20 months old animals reached nearly the limit of their protein accretion capacity and excess energy has been used mainly to increase fat depots. As a dairy breed, Holstein bulls have a lower ability of muscle accretion and a higher preference for fat deposition in comparison to beef breeds^18^. A shortened fattening period to compensate for this breed-related feature was not implemented, which possibly increased the negative effects of the HEP diet on the animals. A changed body mass composition with higher fat content is often associated with negative metabolic consequences. Summarized in various reviews, nutrient excess and obesity can lead to mitochondrial dysfunction, oxidative stress and also membrane and ER stress, which promotes impaired insulin signaling and increased inflammatory cytokine production^19^. Obesity in humans can lead to increased inflammatory mediators^20^, reduced insulin sensitivity or insulin resistance and type 2 diabetes^21,22^. This is similar to observations in horses, where increased body condition score is a predisposition factor for insulin dysregulation and laminitis^23^. Also, overweight cows struggled more to adapt their metabolism to the onset of lactation than lean ones^24,25^.

These associations are consistent with our result that HEP bulls had twice as high insulin concentrations than LEP bulls. In dairy heifers, hyperinsulinemia was defined as > 37.20 µIU/ml^26^. Mean concentration of insulin of IN bulls was 63.59 µIU/ml, and of MO bulls was 30.12 µIU/ml. The results of the studies are consistent, although different assays were used to analyze insulin. However, plasma glucose concentration was not different between feeding groups, which could therefore be termed as compensated insulin resistance. The glucose-to-insulin ratio, as a well-known marker for disturbances in insulin-glucose homeostasis in humans^27,28^, was indicating a decreased insulin sensitivity in HEP bulls. Reduced insulin sensitivity was also observed in heavy veal calves, determined by an euglycemic-hyperinsulinemic clamp^29^ due to intensive feeding.

Blood biochemistry of HEP bulls showed first indications of a potentially tensed metabolic situation due to greater concentrations of urea, phosphate, BHB and the enzyme GLDH. These alterations could indicate higher intake of protein, phosphate and higher production of butyrate in the rumen, which was metabolized to BHB^30^ and a greater protein turn over. Interestingly, there was a remarkable inter-individual variation within the HEP group, especially concentrations of GLDH and BHB were very high in some HEP bulls. Greater concentrations of GLDH could be associated with liver cell damage^31^. Liver abscesses are often observed in feedlot cattle^32^, but were not observed during the routine meat inspection of these bulls. In the rumen of dairy cows, grain-rich diets led to increased concentrations of various potentially toxic metabolites, like methylamines and *N*-nitrosodimethylamines^33^, which could lead to liver cell damage after absorption. Hence, increased concentrations of GLDH could indicate a higher metabolic stress level of the liver in HEP, compared to LEP.

To investigate metabolic pathways in more detail, and in an attempt to understand mechanisms and to generate new hypotheses, we also used a targeted metabolomics approach. The AbsoluteIDQ p180 Kit of Biocrates is specified, inter alia, for inflammation and type 2 diabetes in humans. It quantifies 21 amino acids, 21 biogenic amines, 1 hexose, 40 acylcarnitines, 15 sphingolipids, 14 lyso-PCs and 76 PCs. Especially the last three classes of metabolites seem to be important in the context of inflammation^34–36^. Although the kit was originally developed for human research, it was successfully used in various studies of cattle^15,37–41^.

In line with the results of blood biochemistry, concentrations of arginine, ornithine, and urea were greater in HEP. These metabolites take part in the urea cycle. Protein-rich diets can lead to increased ammonia concentrations in the rumen^42^, excess nitrogen has to be bound in urea during the urea cycle mainly in the liver, to be recycled to the digestive tract or excreted by the kidney^43^. Increased concentrations of these metabolites could also indicate a higher metabolic burden for the liver of HEP bulls.

Glycerophospholipids were found to be the metabolites with the greatest differences between the dietary groups. The class of PC was nearly in total (59 of 76 analyzed PC) lower in HEP. As components of mammalian cell membranes, these lipids influence fluidity of membranes and cell signaling^35,44^. This clear result highlighted the different metabolic states of the dietary groups. Decreased blood concentration of PCs could reflect increased remodeling or degradation of tissues and cells. Various signaling molecules can be generated from different PCs, like diacylglycerol, lyso-PCs, phosphatidic acid and arachidonic acid^36^. As an example, from PC aa/ae 40:4 and chain length above, arachidonic acid (C20:4) and palmitate (C16:0) can be generated. These PCs with long chains were also lower in HEP bulls (Fig. 5g and Supplementary Table S1). So far, there are almost no studies on the individual and exact function of these lipids, but there are several studies which showed alterations of glycerophospholipids in diseases like diabetes^45^ and obesity^21^. Supporting the hypothesis that PCs are needed in case of cell damage and oxidative stress, it was observed in dairy cows around parturition that PC concentrations in blood dramatically decreased and slowly increased again over weeks of early lactation^38,46^. Cell culture studies also showed anti-inflammatory effects^35^ and protective effects of PCs on ethanol-injured hepatocytes, by inhibition of lipid peroxidation^47^.

As mentioned above, PCs can be hydrolyzed by phospholipase A2 to arachidonic acid and lyso-PC. This pathway is activated by inflammatory signals^48^. Decreased concentrations of PCs and increased concentrations of lyso-PCs appeared to be associated with a proinflammatory status. Three lyso-PCs showed higher concentrations in HEP, particularly lyso-PC acyl C18:2 and lyso-PC acyl 14:0 seemed to be firmly associated with inflammation (Fig. 5g). Increased concentrations of these lyso-PCs were also observed as predictive biomarker in diseased cows 4 weeks before parturition in comparison to healthy cows. Diseased cows suffered from mastitis, metritis, retained placenta or laminitis or even combinations of these diseases after parturition^40^. Furthermore, lyso-PC acyl 18:2 was also correlated with insulin resistance in veal calves^29^.

Lower concentrations of SM observed in HEP bulls could reflect a higher consumption for repairing cell damage and can also be linked to a proinflammatory status. Decreased plasma levels of SM were also found in dairy cows around parturition^38,49^, in times of the highest metabolic stress. The enzyme sphingomyelinase can degrade sphingomyelin to ceramide and then to other bioactive lipids, like sphingosine or ceramide-1-phosphate. This pathway can be activated by inflammatory stimuli, like LPS, TNFα or oxidative stress and activates further cytokine production and increased permeability of endothelium^34,50^. Potentially generated ceramides could cause insulin resistance by inhibiting protein kinase B (Akt)^34,51,52^, which was also shown in bovine adipocytes^53^, linking decreased SM and impaired insulin sensitivity. This hypothesis is supported by lower plasma concentrations of some SM in insulin resistant veal calves^29^. Results of the mentioned study was not that clear, but the feeding trial only lasted for 13 weeks and animals were much younger than the animals in this study.

In the compound class of amino acids, especially concentrations of the BCAA and AAA were greater in HEP. Increased plasma concentrations of these amino acids have been associated with obesity, insulin resistance and type 2 diabetes in humans^21,54,55^. Interestingly, increased BCAA concentrations can act as predictive biomarker years before the occurrence of diabetes mellitus^56^. Further, it is known that in case of obesity, gene expression of BCAA catabolic pathway enzymes can be decreased. This could lead to accumulation of toxic amounts of BCAA and their corresponding alpha-ketoacids, further resulting in mitochondrial dysfunction and stress signaling^57^. Reduced activity of BCAA degradation pathway could also be indicated by the result of lower concentration of propionylcarnitine (C3) and other short-chain acylcarnitines in HEP bulls. Short chain acylcarnitines can be derived from the oxidation products of BCAAs^58^. Another aspect is that BCAA can influence innate and adaptive immune system directly and indirectly through activation of mTOR by leucine. BCAA showed effects of upregulation of pro-inflammatory cytokines, immune function of neutrophils and can be taken as fuel sources for immune cells^59^. This could support a proinflammatory status suspected in HEP bulls. Further studies are warranted to confirm all these hypotheses.

**Figure 6:**
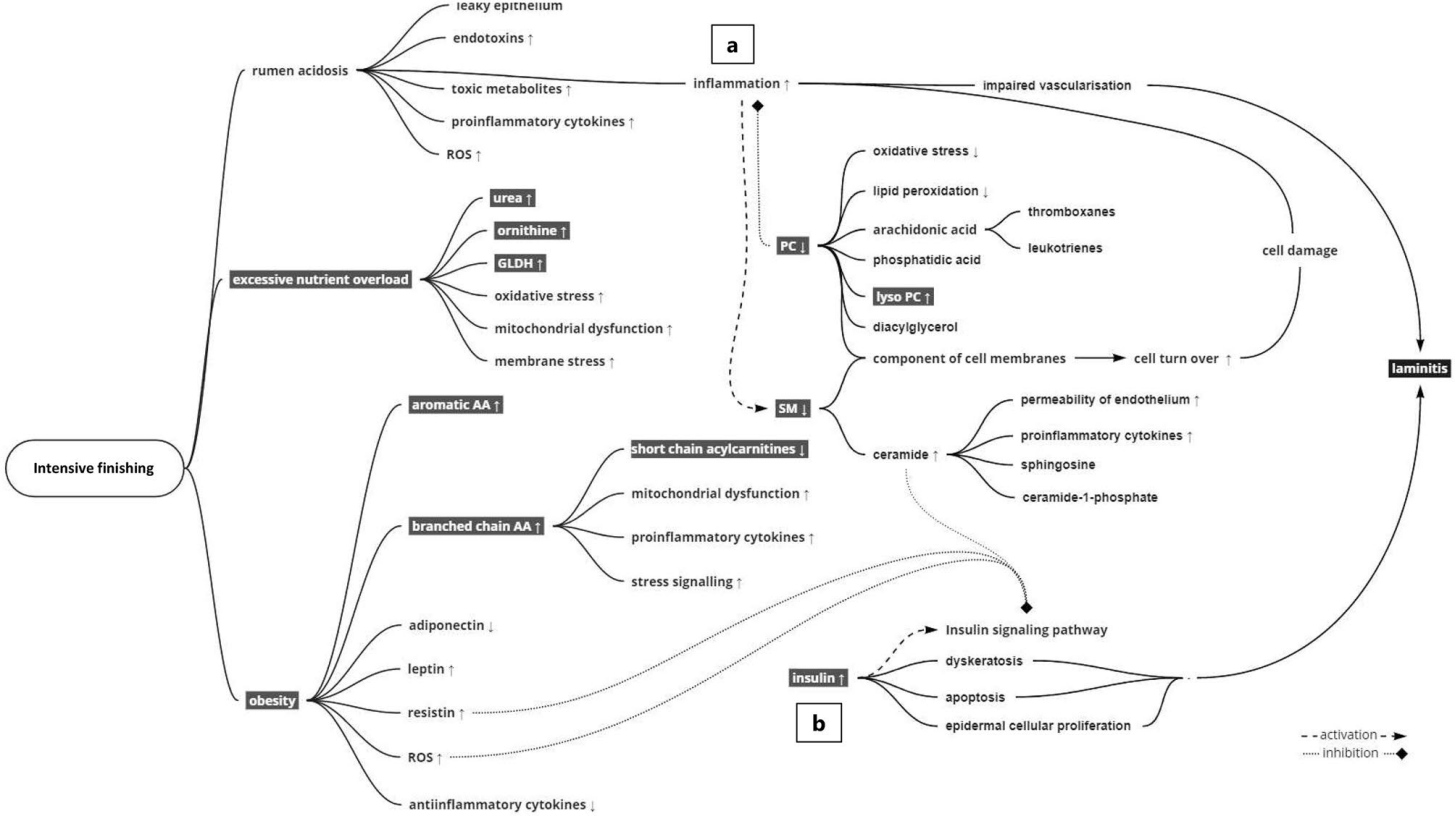
Proposed pathways from intensive finishing towards laminitis. (**a**) Possible alterations related to rumen acidosis, (**b**) possible alterations due to insulin resistance; both potentially causing metabolic inflammation and impaired vascularization which could provoke laminitis. Highlighted factors were observed in Holstein bulls fed high energy and protein diet. Arrows indicate direction of significantly different concentrations in comparison to the group fed low levels of energy and protein. Obesity refers to the documented fat class at slaughter. *ROS* reactive oxygen species, *GLDH* glutamate dehydrogenase, *PC* phosphatidylcholines, *SM* sphingomyelins, *AA* amino acids.

All alterations in the metabolic profile of HEP bulls have in common that they indicate a metabolically tensed or even pathophysiological status of the animals. This is also reflected in the occurrence of the metabolic disorder laminitis, at slaughter only observed in HEP. The chronic excessive nutrient intake likely disturbed metabolic pathways in different ways to such an extent that biological balances could no longer be maintained. A hypothetical connection between the metabolite profiles and laminitis was shown in Fig. 6. There could be two major pathways towards laminitis, which are triggered by excessive nutrient intake and obesity. The first pathway is associated with inflammatory stimuli (Fig. 6a). Starch-rich diet could lead to rumen acidosis with leaky epithelium and increased concentrations of lipopolysaccharides in rumen and blood. Local and systemic inflammation is the consequence. A proinflammatory status is also triggered by obesity-related consequences mentioned above. Inflammation influences activity of many enzymes and transcript abundance of metabolic genes in the liver and thus affects energy metabolism as a whole^60^. Furthermore, inflammation influences vascularization directly and could trigger laminitis through impaired vascularization in the blood vessels of the claws. The second pathway is related to a reduced insulin sensitivity resulting in compensatory hyperinsulinemia (Fig. 6b). Hyperinsulinemia can trigger laminitis in horses, histopathologically shown as dyskeratosis, increased apoptosis and proliferation of the epidermal lamellae in the corium of the hooves^61^. Particularly the combination of obesity, insulin dysregulation and glucose challenge combined are high risk factors for laminitis in horses^62^. This connection is not described in cattle yet. Further research is warranted to verify these hypotheses of laminitis pathogenesis, including time dependent metabolic changes in cattle.

## Conclusion

A wide range of metabolic effects of a chronically increased nutrient (energy, starch and protein) intake was demonstrated by this metabolomics analysis, in an approach to better understand how blood metabolites change due to extreme feeding regimens. Most changes in metabolite profiles of HEP bulls were associated with negative alterations of the metabolism and are a reason for or the consequence of an enhanced metabolic risk to develop inflammatory metabolic disorders, such as laminitis. It can be concluded as a novel hypothesis, that especially lower concentrations of PC and SM and higher concentrations of lyso-PC, BCAA and AAA are associated with an inflammatory state of diet-related laminitis in Holstein fattening bulls. The latter two behave similarly to changes in metabolism of obese humans, indicating a conserved pathophysiological role across different species. Further research is needed to confirm these hypotheses, to understand the pathogenesis of laminitis and connections of metabolic pathways and individual metabolites. These considerations are of great importance not only for the optimal keeping and feeding of fattening bulls and other cattle, but also for nutritional physiology of other species affected by excessive nutrient intake.

## Materials and methods

### Experimental design

The animal experiment was conducted from December 2017 to July 2018 at the Educational and Research Centre for Animal Husbandry, Hofgut Neumuehle, Germany. All experimental procedures were approved by the Animal Ethics Committee of the Department for Animal Welfare Affairs (Landesuntersuchungsamt Rheinland-Pfalz, Koblenz, Germany) in agreement with the German Animal Welfare Act (permit number: G-17-20-070). All procedures were performed in accordance with the approved protocols, relevant guidelines, and regulations. The study was carried out in compliance with the ARRIVE guidelines.

Thirty-two Holstein Friesian bulls were raised with an identical rearing protocol at the Institute of Animal Nutrition, Federal Research Institute for Animal Health (Friedrich-Loeffler-Institut, Braunschweig, Germany). For the feeding trial, bulls were relocated to the Educational and Research Centre for Animal Husbandry (Hofgut Neumuehle, Muenchweiler a.d. Alsenz, Germany). Until the beginning of the experimental trial, bulls were further raised with a total mixed ration (TMR) based on forage and 15% concentrate of DM. Bulls were then randomly assigned to a high energy and protein (HEP) or a low energy and protein (LEP) nutritional regimen, at an average age of 13 months and average body weight of 500 kg (mean ± SD; HEP 506 ± 35 kg, LEP 499 ± 35 kg). They remained on their assigned nutritional regimen for the rest of the study period, 7 months, until slaughter. The diets were formulated according to the highest (HEP) and lowest (LEP) recommendations of the Bavarian State Research Center for Agriculture (LfL)^16^ including ME recommendations for Simmental fattening bulls. Dry matter of the LEP TMR consisted of 100% grass- and corn silage, and the HEP TMR consisted of 54% grass- and corn silage and 46% concentrate. The concentrate feed consisted of ground corn, rapeseed meal, ground wheat, palm kernel meal, wheat bran, molasses, and soybean meal, accounting for an elevated total sugar, total starch and crude protein, as well as a decreased fiber content of the HEP diet, relative to the LEP diet. The exact ingredients and chemical composition of the TMR are given in Tables 1 and 2, respectively. The nutrient composition of the TMR were analyzed by an accredited external laboratory (Landwirtschaftliche Untersuchungs- und Forschungsanstalt, Speyer, Germany) according to the protocols of the Association of German Agricultural Analytic and Research Institutes^63^. The TMR was mixed every 2 days and bulls were fed at 7:30 a.m. daily. Provided feed and remaining feed was weighed and documented group-wise to calculate individual mean feed intake. Bulls were housed on slatted floor with rubber mats, in groups of 4 bulls with a space allowance of 2.9 m^2^/bull.

### Collection of data and blood samples

Live weight was recorded monthly. ADG was calculated from body weight with the exact number of days between weighing. At the beginning of the trial (13 months of age; “baseline”) and at the end (20 months of age; “slaughter) blood samples were taken and health of the claws were documented. Bulls were captured in a hoof trimming chute and health and size of all 4 claws of each animal were documented by trained personnel, using a scoring system according to Sohrt^64^. Length of the dorsal claw wall and thickness of the sole horn of all digits were measured. The following parameters were evaluated as signs of laminitis on the outer and inner digits: (a) concave dorsal claw wall: none = 0¸ low-grade = 1, high-grade = 2; (b) presence of ridging on the dorsal wall of the claw: absent = 0, present = 1; (c) wall defects: none = 0, low-grade = 1, high-grade = 2; (d) double sole: absent = 0, present = 1; (e) discolorations of the sole horn: absent = 0, yellowish = 1, reddish = 2, black-red = 3. Score points were added to one value and maximum score points per foot were 18, and per animal 72. Heel horn erosions were classified and scored on a scale of 0–4 per foot: occurrence on the inner and outer digit: none = 0, low-grade = 1, high-grade = 2. Scoring of interdigital dermatitis was classified as none = 0, superficial = 1, deep = 2. Sole ulcers at typical or atypical localizations were classified as none = 0, discoloration = 1, superficial = 2, deep = 3, perforating = 4. The presence of digital dermatitis, digital phlegmon and tyloma was also evaluated.

Blood samples were collected between 10 a.m. and 2 p.m., by jugular venipuncture into plain tubes for serum, and EDTA tubes and NaF tubes (S-Monovette, Sarstedt AG, Nümbrecht, Germany). The EDTA and NaF samples were centrifuged immediately after sampling at 3000 × *g* for 15 min. Serum samples were allowed to clot for 45 min at 20 °C and then centrifuged at 3000 × *g* for 10 min. Plasma and serum samples were frozen at − 80 °C within 20 min after centrifugation and then continuously stored at − 80 °C until analysis.

A total of 30 animals completed the study; one bull was lost due to sudden death of unidentified cause and one other bull had to be culled before the end of experimental period due to tibial paralysis. Data and already taken samples were not excluded from the study. The 30 bulls that completed the study were slaughtered at an age of 20 months. One day before slaughtering, blood samples were collected from all animals (second, post-treatment samples labeled as “slaughter”), using the same protocol as at baseline. Post mortem measurement and health score of the claws were documented again, with the same protocol which was used at baseline. Conformation and fat class was classified by a trained and experienced member of the slaughterhouse according to the European beef carcass classification system EUROP, where E is Excellent, U is Very Good, R is Good, O is Fair and P is Poor muscle development. Fat class is differentiated into 5 classes, where 1 is Low, 2 is Slight, 3 is Average, 4 is High, and 5 is Very High amount of fat covering. Making the scoring blinded was not possible. Conformation classes were transformed to class numbers (Poor = 1—Excellent = 5) for statistical analyses.

### Blood metabolite, hormone, and metabolome analyses

At the time of slaughter, glucose and l-lactate in NaF plasma and urea, creatinine, total protein, albumin, aspartate aminotransferase, gamma-glutamyl transferase, glutamate dehydrogenase, creatine kinase, phosphate, beta-hydroxybutyrate and non-esterified fatty acids in serum were analyzed with UV or visible colorimetric spectroscopy based enzymatic assays on an automated analyzer (cobas c311 Analyzer, ROCHE, Mannheim, Germany). Insulin was measured in serum by a sandwich-ELISA (Bovine Insulin ELISA, 10-1201-01, Mercodia AB, Uppsala, Sweden) according to the manufacturer’s protocol at baseline and slaughter timepoints.

Metabolomics analysis was performed using EDTA plasma at baseline and slaughter. The metabolome analysis was carried out using the AbsoluteIDQ p180 Kit (Biocrates Life Science AG, Innsbruck, Austria) according to the manufacturer´s protocol with modifications pointed out below. This kit identifies and quantifies up to 188 metabolites from 5 compound classes: acylcarnitines (40), proteinogenic and modified amino acids (19), glycerophospho- and sphingolipids (76 phosphatidylcholines, 14 lysophosphatidylcholines, 15 sphingomyelins), biogenic amines (19) and hexoses (1). The amino acids and biogenic amines were analyzed by liquid chromatography–mass spectrometry (LC–MS/MS) and the substance classes of acylcarnitines, phosphatidylcholines (including lyso-phosphatidylcholines), sphingomyelins and hexoses were analyzed by flow injection analysis—mass spectrometry (FIA-MS/MS) at the Core Facility of the University of Hohenheim (Stuttgart, Germany). Internal standard, PBS (phosphate buffer saline), calibration standards (“Cal 0.25”, “Cal 0.5”, Cal 1–6), quality control samples (QC 1–3) and EDTA plasma samples (10 µL) were applied and dried onto the matrix of the multititer plate provided in the kit under nitrogen flow (nitrogen evaporator 96 well plate, VLM GmbH, Bielefeld, Germany) for 30 min. “Cal 0.25” and “Cal 0.5” were 4 × and 2 × dilutions of the lowest calibrator solution included in the kit, respectively. These were used to enhance accuracy in the lower detection range, according to consultation with the manufacturer. The rest of the analytical procedures were carried out analogously to previous measurements described in Kenéz et al.^38^. Dried samples were derivatized with 5% phenylisothiocyanate (PITC) for 20 min at room temperature and subsequently dried for another 60 min under nitrogen flow. Samples were extracted in 300 µL of extraction solvent (5 mM ammonium acetate in methanol) with shaking at 450 rpm for 30 min at room temperature. Eluted extracts were 10 × diluted in 40% HPLC grade methanol for LC–MS analysis and 50 × diluted in the provided mobile phase solvent for FIA-MS/MS analysis. Both types of measurements were performed on a QTRAP mass spectrometer applying electrospray ionization (ESI) (ABI Sciex API 5500Q-TRAP). The MS was coupled to an ultra-performance liquid chromatography (UPLC) (Agilent 1290, Agilent, Waldbronn, Germany). In case of LC–MS the metabolites were separated by a hyphenated reverse phase column (Waters, ACQUITY BEH C18, 2.1 × 75 mm, 1.7 µm; Waters, Milford, United States) preceded with a precolumn (Security Guard, Phenomenex, C18, 4 9 3 mm; Phenomenex, Aschaffenburg, Germany) applying a gradient of solvent A (formic acid 0.2% in water) and solvent B (formic acid 0.2% in acetonitrile) over 7.3 min (0.45 min 0% B, 3.3 min 15% B, 5.9 min 70% B, 0.15 min 70% B, 0.5 min 0% B) at a flow rate of 800 µL/min. Oven temperature was 50 °C. For LC–MS analysis 5 µL, and for FIA 20 µl were subjected for measurements in both positive and negative mode. Identification and quantification were achieved by multiple reaction monitoring (MRM) standardized by applying spiked-in isotopically labelled standards in both positive and negative mode. A calibrator mix consisting of eight different concentrations was used for calibration. Quality controls deriving from lyophilized human plasma samples were included for 3 different concentration levels. For FIA an isocratic method was used (100% organic running solvent) with varying flow conditions (0 min, 30 µL/min; 1.6 min 30 µL/min; 2.4 min, 200 µL/min; 2.8 min, 200 µL/min; 3 min 30 µL/min), and the MS settings were as follows: scan time 0.5 s, IS voltage for positive mode 5500 V, for negative mode − 4500 V, source temperature 200 °C, nitrogen as collision gas medium; the corresponding parameters for LC–MS were: scan time 0.5 s, source temperature 500 °C, nitrogen as collision gas medium). All reagents used in the processing and analysis were of LC–MS grade, unless otherwise stated. Milli-Q Water ultrapure was used fresh after being prepared by the high-purity water system by Merck KGaA (Darmstadt, Germany). LC–MS grade acetonitrile (83640.3201, VWR), Water (83645.320, VWR) and methanol (1.000971.500), as well as pyridine for analysis (1.09728.0100) and formic acid (98–100%; 1.000263.1000) were purchased by Merck KGaA. PITC (P10034-10) and ammoniumacetate (81.7838-50) were purchased by Sigma Aldrich Chemie GmbH (Steinheim, Germany). Raw data of the Analyst software (AB Sciex, Framingham, MA, USA) were processed by the MetIDQ software which is an integrated part of the p180 Kit (Biocrates Life Sciences AG). This streamlines data analysis by automated calculation of metabolite concentrations providing quality measures and quantification^38^. Before integrating LC–MS data in MetIDQ, it was validated in Analyst software. For fully quantitative measurements of the p180 Kit, the lower limit of quantification (LLOQ) was determined in plasma experimentally by the manufacturer.

### Data analysis and visualization

Metabolites were excluded from the analyses if ≥ 70% of samples were 0 µmol/L. Following metabolites were excluded: spermine, phenylethylamine, dopamine, dihydroxyphenylalanine, *cis*-4-hydroxyproline and sphingomyelin 22:3. Data of the remaining 182 plasma metabolites [absolute concentrations (µmol/L) of compounds] were analyzed in MetaboAnalyst 4.0^65^ after normalization by log transformation and Pareto scaling. The functions used were: principal component analysis (PCA), to depict how variation in the data was distributed across the sampling days and to show the variation between the animals, and sparse partial least squares discriminant analysis (sPLS-DA) to identify which compounds were most accountable for the variation between the dietary treatment groups. The sPLS-DA model was quality checked by cross validation. Furthermore, a heatmap was created to visualize differences between the dietary groups at slaughter. In addition, JMP.Pro 15 (SAS Institute, Cary, NC, USA) was used to detect significant differences between the groups by unpaired Student’s t-test of fat class, conformation class and metabolites [including glucose and glucose-to-insulin (G:I) ratio] at slaughter. The G:I ratio was calculated according to Legro^27^. Repeated measure two-way ANOVA was performed to detect effects of time, diet and time × diet interaction on concentration of insulin and score points of laminitis and heel horn erosion, length and thickness of the claws, feed intake, body weight and ADG. Significant interactions were afterwards checked by HSD Tukey post-hoc test. Data of laminitis and heel horn erosion were normalized by log transformation before performing the ANOVA. Nominal logistic regression was used to detect effects of time, group and time × group interaction on prevalence of claw diseases, tested by Likelihood ratio tests. Differences in prevalence of claw diseases between groups and timepoints were tested by Fisher’s exact test. Level of significance was set at P < 0.05. Important findings were visualized with bar charts performed in JMP.Pro 15.

## Supplementary Information

Supplementary Table S1.

## Data Availability

Original data are available from the corresponding author upon reasonable request.
