# Supplementary material for: Association between alterations in plasma metabolome profiles and laminitis in intensively finished Holstein bulls in a randomized controlled study
Source: Sci Rep. 2021 Jun 17;11:12735. doi: 10.1038/s41598-021-92163-6 (PMC8211646; doi:10.1038/s41598-021-92163-6)
Supplement: Supplementary file 1 — Supplementary Table S1. [file 41598_2021_92163_MOESM1_ESM.pdf]

Association between alterations in plasma metabolome profiles and laminitis in intensively finished Holstein bulls: a randomized controlled study

Sonja Christiane Bäßler, Ákos Kenéz, Theresa Scheu, Christian Koch, Ulrich Meyer, Sven Dänicke, Korinna Huber

SUPPLEMENTARY MATERIAL

Table S1. Significantly different metabolites between bulls fed a high energy and protein (HEP) or a low energy and protein (LEP) diet, and all results of measured serum biochemistry. SM = sphingomyelin, PC = phosphatidylcholine, aa = diacyl, ae = acyl-alkyl.

	HEP	LEP	units	p-value
**Arginine**	195.0 ± 6.7	175.3 ± 6.3	µmol/L	< **0.05**
**Asparagine**	38.9 ± 2.2	32.2 ± 1.4	µmol/L	< **0.05**
**Isoleucine**	136.8 ± 5.8	103.0 ± 5.1	µmol/L	< **0.001**
**Leucine**	164.8 ± 9.7	113.0 ± 6.1	µmol/L	< **0.001**
**Lysine**	142.5 ± 8.3	107.4 ± 4.8	µmol/L	< **0.01**
**Ornithine**	60.3 ± 2.5	47.6 ± 1.7	µmol/L	< **0.001**
**Phenylalanine**	55.8 ± 2.9	45.1 ± 1.7	µmol/L	< **0.01**
**Threonine**	75.0 ± 5.4	52.6 ± 1.9	µmol/L	< **0.001**
**Tryptophan**	54.0 ± 3.1	46.1 ± 1.9	µmol/L	< **0.05**
**Tyrosine**	68.2 ± 3.2	58.2 ± 1.9	µmol/L	< **0.05**
**Valine**	259.3 ± 11.5	199.3 ± 7.7	µmol/L	< **0.001**
**Acetylornithine**	2.3 ± 0.2	3.8 ± 0.3	µmol/L	< **0.001**
**Creatinine**	67.4 ± 3.8	83.0 ± 2.2	µmol/L	< **0.01**
***Trans*-4-Hydroxyproline**	28.1 ± 1.0	32.3 ± 1.4	µmol/L	< **0.05**
**lyso PC a C14:0**	4.4 ± 0.1	4.1 ± 0.1	µmol/L	< **0.05**
**lyso PC a C16:0**	17.9 ± 0.9	14.2 ± 0.6	µmol/L	< **0.01**
**lyso PC a C18:2**	14.0 ± 0.7	10.5 ± 0.4	µmol/L	< **0.001**
**SM (OH) C14:1**	7.4 ± 0.3	10.6 ± 0.6	µmol/L	< **0.001**
**SM (OH) C16:1**	6.2 ± 0.3	8.2 ± 0.5	µmol/L	< **0.001**
**SM (OH) C22:2**	4.2 ± 0.2	5.4 ± 0.3	µmol/L	< **0.01**
**SM C18:0**	9.6 ± 0.5	11.7 ± 0.6	µmol/L	< **0.05**
**SM C18:1**	4.0 ± 0.2	4.8 ± 0.2	µmol/L	< **0.05**
**SM C24:0**	18.2 ± 1.2	31.7 ± 1.6	µmol/L	< **0.001**
**SM C26:0**	0.28 ± 0.02	0.36 ± 0.03	µmol/L	< **0.05**
**C3**	0.36 ± 0.02	0.46 ± 0.04	µmol/L	< **0.05**
**C3-OH**	0.02 ± 0.00	0.02 ± 0.00	µmol/L	< **0.05**
**C5-M-DC**	0.03 ± 0.00	0.03 ± 0.00	µmol/L	< **0.01**

**C6:1**	0.03 ± 0.00	0.04 ± 0.00	µmol/L	< **0.05**
**C12:1**	0.11 ± 0.00	0.09 ± 0.01	µmol/L	< **0.01**
**C6 (C4:1 DC)**	0.06 ± 0.00	0.08 ± 0.01	µmol/L	< **0.05**
**PC aa C30:2**	0.1 ± 0.0	0.2 ± 0.2	µmol/L	< **0.01**
**PC aa C32:0**	4.4 ± 0.2	5.1 ± 0.2	µmol/L	< **0.01**
**PC aa C32:1**	4.6 ± 0.2	5.3 ± 0.2	µmol/L	< **0.05**
**PC aa C32:2**	4.2 ± 0.3	6.0 ± 0.4	µmol/L	< **0.001**
**PC aa C34:2**	109.7 ± 6.0	85.3 ± 3.7	µmol/L	< **0.01**
**PC aa C34:3**	16.2 ± 1.0	23.7 ± 1.3	µmol/L	< **0.001**
**PC aa C36:0**	4.1 ± 0.3	10.0 ± 1.0	µmol/L	< **0.001**
**PC aa C36:1**	94.7 ± 4.2	133.4 ± 5.5	µmol/L	< **0.05**
**PC aa C36:3**	57.2 ± 2.3	71.2 ± 11.7	µmol/L	< **0.01**
**PC aa C36:4**	19.9 ± 1.1	23.9 ± 1.2	µmol/L	< **0.05**
**PC aa C36:5**	4.2 ± 0.2	6.4 ± 0.4	µmol/L	< **0.001**
**PC aa C36:6**	1.3 ± 0.1	1.9 ± 0.1	µmol/L	< **0.01**
**PC aa C38:0**	1.5 ± 0.1	4.2 ± 0.3	µmol/L	< **0.001**
**PC aa C38:1**	2.8 ± 0.2	9.7 ± 0.6	µmol/L	< **0.001**
**PC aa C38:3**	28.2 ± 2.0	38.4 ± 2.0	µmol/L	< **0.01**
**PC aa C38:4**	34.9 ± 30.6	44.4 ± 2.1	µmol/L	< **0.01**
**PC aa C38:5**	11.5 ± 0.7	18.6 ± 1.3	µmol/L	< **0.001**
**PC aa C38:6**	2.2 ± 0.1	3.4 ± 0.2	µmol/L	< **0.001**
**PC aa C40:1**	0.2 ± 0.0	0.3 ± 0.0	µmol/L	< **0.001**
**PC aa C40:2**	0.2 ± 0.0	0.5 ± 0.0	µmol/L	< **0.001**
**PC aa C40:3**	2.4 ± 0.2	5.8 ± 0.5	µmol/L	< **0.001**
**PC aa C40:4**	9.1 ± 0.8	18.9 ± 1.3	µmol/L	< **0.001**
**PC aa C40:5**	13.3 ± 0.8	20.9 ± 1.2	µmol/L	< **0.001**
**PC aa C40:6**	3.6 ± 0.3	6.3 ± 0.5	µmol/L	< **0.001**
**PC aa C42:0**	0.06 ± 0.00	0.07 ± 0.00	µmol/L	< **0.001**
**PC aa C42:1**	0.06 ± 0.00	0.09 ± 0.00	µmol/L	< **0.001**
**PC aa C42:2**	0.09 ± 0.00	0.11 ± 0.00	µmol/L	< **0.001**
**PC aa C42:4**	0.2 ± 0.0	0.3 ± 0.0	µmol/L	< **0.001**
**PC aa C42:5**	0.6 ± 0.0	2.3 ± 0.2	µmol/L	< **0.001**
**PC aa C42:6**	0.3 ± 0.0	1.1 ± 0.1	µmol/L	< **0.001**
**PC ae C30:0**	0.37 ± 0.02	0.44 ± 0.01	µmol/L	< **0.05**
**PC ae C32:1**	1.7 ± 0.1	2.1 ± 0.1	µmol/L	< **0.01**
**PC ae C34:0**	1.9 ± 0.1	2.8 ± 0.1	µmol/L	< **0.001**
**PC ae C34:1**	9.1 ± 0.4	12.8 ± 0.6	µmol/L	< **0.001**
**PC ae C34:2**	12.2 ± 0.7	16.4 ± 0.8	µmol/L	< **0.001**
**PC ae C36:0**	1.5 ± 0.1	2.8 ± 0.2	µmol/L	< **0.001**
**PC ae C36:1**	11.9 ± 0.5	18.2 ± 0.9	µmol/L	< **0.001**
**PC ae C36:2**	17.8 ± 0.8	20.8 ± 0.9	µmol/L	< **0.05**
**PC ae C36:3**	6.8 ± 0.3	9.3 ± 0.5	µmol/L	< **0.001**
**PC ae C38:0**	1.0 ± 0.1	2.1 ± 0.1	µmol/L	< **0.001**
**PC ae C38:1**	1.6 ± 0.1	3.2 ± 0.2	µmol/L	< **0.001**
**PC ae C38:2**	2.4 ± 0.1	3.6 ± 0.2	µmol/L	< **0.001**
**PC ae C38:3**	2.7 ± 0.1	3.6 ± 0.2	µmol/L	< **0.001**

**PC ae C38:4**	3.6 ± 0.2	5.1 ± 0.2	µmol/L	< **0.001**
**PC ae C38:5**	2.7 ± 0.2	3.3 ± 0.2	µmol/L	< **0.05**
**PC ae C40:1**	0.2 ± 0.0	0.4 ± 0.0	µmol/L	< **0.001**
**PC ae C40:2**	0.7 ± 0.0	1.0 ± 0.0	µmol/L	< **0.001**
**PC ae C40:3**	0.7 ± 0.0	0.9 ± 0.0	µmol/L	< **0.001**
**PC ae C40:4**	0.9 ± 0.0	1.2 ± 0.1	µmol/L	< **0.001**
**PC ae C40:5**	1.3 ± 0.1	2.0 ± 0.1	µmol/L	< **0.001**
**PC ae C40:6**	0.7 ± 0.0	1.0 ± 0.0	µmol/L	< **0.01**
**PC ae C42:1**	0.1 ± 0.0	0.2 ± 0.0	µmol/L	< **0.001**
**PC ae C42:2**	0.15 ± 0.01	0.22 ± 0.01	µmol/L	< **0.001**
**PC ae C42:3**	0.1 ± 0.0	0.2 ± 0.0	µmol/L	< **0.001**
**PC ae C42:4**	0.1 ± 0.0	0.2 ± 0.0	µmol/L	< **0.001**
**PC ae C42:5**	0.5 ± 0.0	0.7 ± 0.0	µmol/L	< **0.001**
**PC ae C44:3**	0.07 ± 0.00	0.08 ± 0.00	µmol/L	< **0.001**
**PC ae C44:4**	0.08 ± 0.00	0.1 ± 0.00	µmol/L	< **0.01**
**PC ae C44:5**	0.07 ± 0.00	0.09 ± 0.00	µmol/L	< **0.001**
**PC ae C44:6**	0.06 ± 0.00	0.07 ± 0.00	µmol/L	< **0.001**
**Insulin**	3.1 ± 0.2	1.5 ± 0.2	µg/L	< **0.001**
**Glucose**	4.1 ± 0.2	4.2 ± 0.1	mmol/L	0.794
**L-Lactate**	1.5 ± 0.3	2.2 ± 0.5	mmol/L	0.245
**Urea**	5.0 ± 0.1	2.8 ± 0.1	mmol/L	< **0.001**
**Creatinine**	73.5 ± 2.2	86.9 ± 2.2	µmol/L	< **0.001**
**Total protein**	69.9 ± 3.1	68.8 ± 0.4	g/L	0.201
**Albumin**	39.7 ± 0.4	39.3 ± 0.4	g/L	0.333
**gamma Glutamyltransferase**	16.1 ± 1.4	14.5 ± 1.0	U/L	0.27
**Aspartate aminotransferase**	86.8 ± 3.1	83.1 ± 2.1	U/L	0.328
**Glutamate dehydrogenase**	16.0 ± 2.3	10.1 ± 0.9	U/L	< **0.05**
**Creatine kinase**	230.8 ± 50.2	176.6 ± 7.5	U/L	0.295
**Phosphate**	2.3 ± 0.0	2.2 ± 0.0	mmol/L	< **0.05**
**beta-Hydroxybutyrate**	0.5 ± 0.0	0.4 ± 0.0	mmol/L	< **0.05**
**Non-esterified fatty acids**	0.12 ± 0.01	0.12 ± 0.01	mmol/L	0.905
